# Identification of subtypes of hepatocellular carcinoma and screening of prognostic molecular diagnostic markers based on cell adhesion molecule related genes

**DOI:** 10.3389/fgene.2022.1042540

**Published:** 2022-11-22

**Authors:** Ruge Sun, Yanchao Gao, Fengjun Shen

**Affiliations:** ^1^ College of Medicine, Shanxi Medical University, Taiyuan, China; ^2^ Department of Gastroenterology and Hepatoloy, The First Hospital of Shanxi Medical University, Taiyuan, China; ^3^ Department of Hepatobiliary Surgery, Liaocheng People’s Hospital, Liaocheng, China

**Keywords:** liver hepatocellular carcinoma, cell adhesion molecules, nonnegative matrix factorization (NMF), cluster, risk model, functional enrichment analysis, immune infiltration, bioinformatics

## Abstract

Cell adhesion molecules can predict liver hepatocellular carcinoma (LIHC) metastasis and determine prognosis, while the mechanism of the role of cell adhesion molecules in LIHC needs to be further explored. LIHC-related expression data were sourced from The *Cancer* Genome Atlas (TCGA) and the gene expression omnibus (GEO) databases, and genes related to cell adhesion were sourced from the Kyoto Encyclopedia of Genes and Genomes (KEGG) database. First, the TCGA-LIHC dataset was clustered by the nonnegative matrix factorization (NMF) algorithm to find different subtypes of LIHC. Then the difference of prognosis and immune microenvironment between patients of different subtypes was evaluated. In addition, a prognostic risk model was obtained by least shrinkage and selection operator (LASSO) and Cox analysis, while a nomogram was drawn. Furthermore, functional enrichment analysis between high and low risk groups was conducted. Finally, the expressions of model genes were explored by quantitative real-time polymerase chain reaction (qRT-PCR). The 371 LIHC patients were classified into four subtypes by NMF clustering, and survival analysis revealed that disease-free survival (DFS) of these four subtypes were clearly different. Cancer-related pathways and immune microenvironment among these four subtypes were dysregulated. Moreover, 58 common differentially expressed genes (DEGs) between four subtypes were identified and were mainly associated with PPAR signaling pathway and amino acid metabolism. Furthermore, a prognostic model consisting of IGSF11, CD8A, ALCAM, CLDN6, JAM2, ITGB7, SDC3, CNTNAP1, and MPZ was built. A nomogram consisting of pathologic T and riskScore was built, and the calibration curve illustrated that the nomogram could better forecast LIHC prognosis. Gene Set Enrichment Analysis (GSEA) demonstrated that DEGs between high and low risk groups were mainly involved in cell cycle. Finally, the qRT-PCR illustrated the expressions of nine model genes between normal and LIHC tissue. A prognostic model consisting of IGSF11, CD8A, ALCAM, CLDN6, JAM2, ITGB7, SDC3, CNTNAP1, and MPZ was obtained, which provides an important reference for the molecular diagnosis of patient prognosis.

## Introduction

Liver cancer is a common malignant tumor of digestive system all over the world (Zhou et al., 2016). According to the latest data updated by GLOBOCAN 2020, the number of new cases of liver cancer in the world has reached 905,600, ranking sixth in malignant tumors, and 830,200 deaths (Sung et al., 2021), ranking third in malignant tumors (Gao et al., 2021). Hepatocellular carcinoma (LIHC) accounts for 85%–90% of primary liver cancer. LIHC mainly originates from chronic liver injury (i.e. chronic viral hepatitis B or hepatitis C or cirrhosis caused by long-term drinking) (Arnold et al., 2020). The most effective treatment for LIHC is surgical resection, followed by ablation, radiotherapy, immunotherapy, liver transplantation, chemotherapy, targeted therapy and so on (Zhou and Song 2021). However, the results are not very satisfactory. About 70% of LIHC patients have tumor recurrence within 5 years after curative resection or ablation. In China, the 5-year survival rate of LIHC patients was only 14.1% (Cao et al., 2021). The overall adverse outcome can be attributed to the fact that patients are already in an advanced stage at the time of diagnosis, of which less than 30% can be operated on. Therefore, in order to improve the prognosis and survival rate of patients, it is urgent to explore prognostic molecular diagnostic markers and establish prognostic molecular models of LIHC.

Cell adhesion molecules are collectively referred to as many molecules that mediate the contact and binding between cells or between cells and extracellular matrix (Gibson 2011). Adhesion molecules play a role in the form of receptor ligand binding, which makes cells adhere to each other, between cells and matrix, or between cells matrix cells. They participate in cell recognition, cell activation and signal transduction, cell proliferation and differentiation, cell extension, and movement. Many studies have shown that cell adhesion molecules were important in physiopathological processes such as immune response, inflammation, coagulation, tumor metastasis, and wound healing. For example, ICAM-1 is a cell surface glycoprotein and it can transfer leukocyte recruitment from circulation to sites of inflammation (Moore and Hinni 2013). It has been reported that ICAM-1 is a marker of LIHC stem cells in humans and mice and ICAM-1 inhibitors could slow tumor formation and metastasis in mice (Liu et al., 2013). Metallothionein has been proved to be related to tumor staging, treatment resistance, poor prognosis and survival rate of many cancers. Khan t mentioned that CDCP1(Heitmann et al., 2020), a transmembrane protein, was significantly up-regulated in LIHC(Khan et al., 2021).

In this study, based on the expression of 146 cell adhesion factor related genes, we combined the Non-negative Matrix Factorization (NMF) algorithm to perform consistent clustering on TCGA patient samples, and found four subtypes to identify liver cancer diseases. In order to observe the immune status of various disease subtypes, we analyzed the immune infiltration of tumor patients’ tissues and observed the distribution of immune cells through ESTIMATE, CIBERSORT and ssGSEA. In addition, we obtained a prognostic molecular diagnostic model composed of nine genes through univariate Cox analysis and LASSO regression analysis. At the same time, we combined the clinical information of patients to conduct independent prognostic analysis and nomogram and calibration curve drawing. In general, our prognostic molecular diagnostic model has good diagnostic significance for the prognosis of patients, and provides an important reference for the molecular diagnosis of the prognosis of LIHC patients in the future.

## Materials and methods

### Data source

We extracted the LIHC-related expression data (LIHC = 371, normal = 50) and its clinical data from The *Cancer* Genome Atlas (TCGA, https://portal.gdc.cancer.gov/) database (accessed on 15 April 2022). Moreover, the GSE76427 dataset (LIHC = 115, normal = 52) was obtained from the Gene Expression Omnibus (GEO) database (https://www.ncbi.nlm.nih.gov/geo/). Moreover, a total of 146 genes relevant to cell adhesion molecules pathway (hsa04514) were downloaded from the Kyoto Encyclopedia of Genes and Genomes (KEGG) database.

### Nonnegative matrix factorization clustering analysis

In order to investigate whether cell adhesion genes are associated with the development of LIHC, we performed a cluster analysis of 371 LIHC samples from TCGA-LIHC by “NMF” R package based on 146 cell adhesion molecules-related genes, and classified the LIHC samples into different disease subtypes. Then, we plotted a heat map of the expression of cell adhesion molecules-related genes in each subtype. Next, according to the survival status of LIHC, the survival curves of patients of each subtype were plotted by “survminer” R package. Finally, KEGG pathway enrichment analysis was performed by “GSVA” R package (Hanzelmann et al., 2013) to observe the changes of metabolic pathways among patients of each subtype.

### Analysis of the immune microenvironment among different subtypes

Immune changes in the body are important in the evolution of cancer and the anti-tumor process (Gajewski et al., 2013). To assess the immune profile among disease subtypes, we performed immune infiltration analysis by ESTIMATE, Cell type Identification By Estimating Relative Subsets Of RNA Transcripts (CIBERSORT), and single sample gene set enrichment analysis (ssGSEA), respectively. First, “ESTIMATE” R package assessed stromal cell score, immune cell score, and tumor purity, and the distribution of each score among subtypes of patients was compared by “ggpubr” R package combined with analysis of variance (ANOVA) test. CIBERSORT was able to quantify the relative score of 22 immune cell. In this analysis, the proportion distribution of immune cell was obtained by CIBERSORT and compared between groups by the wilcox test. To further understand the distribution of immune cells among subtypes, the content of 28 immune cells was assessed by “GSVA” R package (Hanzelmann et al., 2013). Also, the differences between groups were analyzed by ANOVA test.

### Identification of differentially expressed genes (DEGs) among different subtypes

To explore the differential expression of genes among subtypes, the DEGs between multiple groups were gotten using “limma” R package combined with F test (Cluster 2 vs. Cluster 1, Cluster 3 vs. Cluster 1, Cluster 4 vs. Cluster 1, Cluster 3 vs Cluster 2, Cluster 4 vs. Cluster 3, Cluster 4 vs. Cluster 3), with a screening threshold of |log_2_ (fold change)| ≥ 0.5 and *p* < 0.05. The DEGs between different subtypes were then intersected to obtain the common DEGs. Then, in order to observe the functions and pathways involved in the common DEGs, gene ontology (GO) and KEGG enrichment analyses were performed by “clusterProfiler” R package on the common DEGs.

### Building and validation of a risk model

To observe whether cell adhesion molecules-related genes have diagnostic potential for survival prognosis, 146 cell adhesion molecules-related genes were screened for risk factors in the TCGA-LIHC dataset by univariate Cox analysis with the threshold set to HR ≥ 1.1 or HR < 0.9 and *P* ≤ 0.05. Then least shrinkage and selection operator (LASSO) regression analysis was conducted using the “glmnet” R package combined with the expression of key univariate candidate risk factors (family = “cox”, nfolds = 10) to obtain the signature genes. Then, the signature genes obtained from LASSO regression analysis were used as input data for multivariate Cox analysis combined with STEP method. Combining the multivariate Cox and the expression of the model genes, the risk score was gotten by the predict. coxph function with the following formula.

$$risk\;score=\sum_{n=1}^{n}\left({coef}_{i}\times x_{i}\right)$$

The optimal threshold analysis was also performed, and 365 patients (excluding six samples with incomplete clinical information) were grouped into high- and low-risk groups. To observe the survival of patients between the high- and low-risk groups, survival analysis was performed, and survival curves were plotted. Receiver Operating Characteristic (ROC) curves were also plotted to determine the diagnostic efficiency of the risk model. In addition, to ensure the reliability and reproducibility of the constructed risk model, the GSE76427 dataset was selected for validation.

### Independent prognostic analysis and building of a nomogram

To clarify if the risk model was an independent prognostic factor relative to other clinical characteristics, the risk model was subjected to univariate Cox analysis in combination with clinical characteristics such as subtype, age, tumor pathological stage, and gender. Then the prognostic factors that were significant in the univariate Cox model were subjected to multivariate Cox analysis. Ultimately, a nomogram and calibration curves for clinical diagnosis were plotted in combination with the results of the Cox analysis.

### The gene set enrichment analysis (GSEA)

To explore the changes in pathways between high and low risk groups, DEGs were screened by “limma” R package (Ritchie et al., 2015) and GSEA analysis was conducted on DEGs using “GSVA” R package (Hanzelmann (Hanzelmann et al., 2013).

### Quantitative real-time polymerase chain reaction

Six LIHC patients were recruited from Liaocheng People’s Hospital (Liaocheng, Shandong Province, China), and LIHC tissue and paracancerous tissue from LIHC patients were collected. All LIHC cases endorsed informed consent forms and the study passed the ethical review of Liaocheng People’s Hospital. First, total RNA was extracted by TRIzol Reagent from ambion company, Inc. Then, reverse transcription reaction was performed by SureScript First strand cDNA synthesis kit provided by the Servicebio company. PCR was conducted using the 2xUniversal Blue SYBR Green qPCR Master Mix kit provided by Servicebio. The PCR conditions were: 95°C pre-denaturation for 1 min, and then 40 cycles. Each cycle included 95°C denaturation for 20s, 55°C annealing for 20 s, and 72°C extension for 30 s. GAPDH was used as an internal reference for gene detection. Primer sequences were shown in Table 1. The expressions of model genes in LIHC tissue and paracancerous tissue were compared by ANOVA.

**TABLE 1:** Primers used for reverse transcription-quantitative PCR.

	Sequence
JAM2 F	ATT​TTA​GCC​TGC​AAA​ACC​CCA
JAM2 R	AAC​GAT​ATT​TCC​CCG​CAT​CAC
IGSF11 F	TAT​CAG​GGT​GGA​CAG​ATG​TTT​GA
IGSF11 R	TTG​GAG​GTA​GTT​TGA​GGG​TAT​TG
CD8A F	CGACTTCCGCCGAGAGAA
CD8A R	CACAGGCCGGGGACATTT
ALCAM F	CAG​CAG​AAA​ACC​AAC​TGG​AGA​G
ALCAM R	CAG​CAA​GGA​GGA​GAC​CAA​CAA​C
CLDN6 F	GCC​TTT​TGT​TGC​TGG​GTG​G
CLDN6 R	GAT​GGC​AGG​GGC​AGA​TGT​T
ITGB7 F	GAG​GGT​AAG​GCT​GAG​GAT​CG
ITGB7 R	AGT​GGG​TGG​CTT​GGA​GAG​AA
SDC3 F	ACA​CAA​CCA​GAC​ACA​GCC​AAT​G
SDC3 R	GTG​ACC​AAG​AAG​GCA​GCA​AAG​A
CNTNAP1 F	GCT​TCT​CCT​TTT​CTC​CCG​TC
CNTNAP1 R	CTC​TGC​CCC​TTC​CAC​ATC​AT
MPZ F	TAG​AAC​TCC​TCC​GCA​ACC​G
MPZ R	CAA​AAC​CAA​GCC​CAC​CAC​C
GAPDH F	CCC​ATC​ACC​ATC​TTC​CAG​G
GAPDH R	CAT​CAC​GCC​ACA​GTT​TCC​C

## Results

### NMF clustering can classify LIHC patients into different subtypes

**FIGURE 1:**
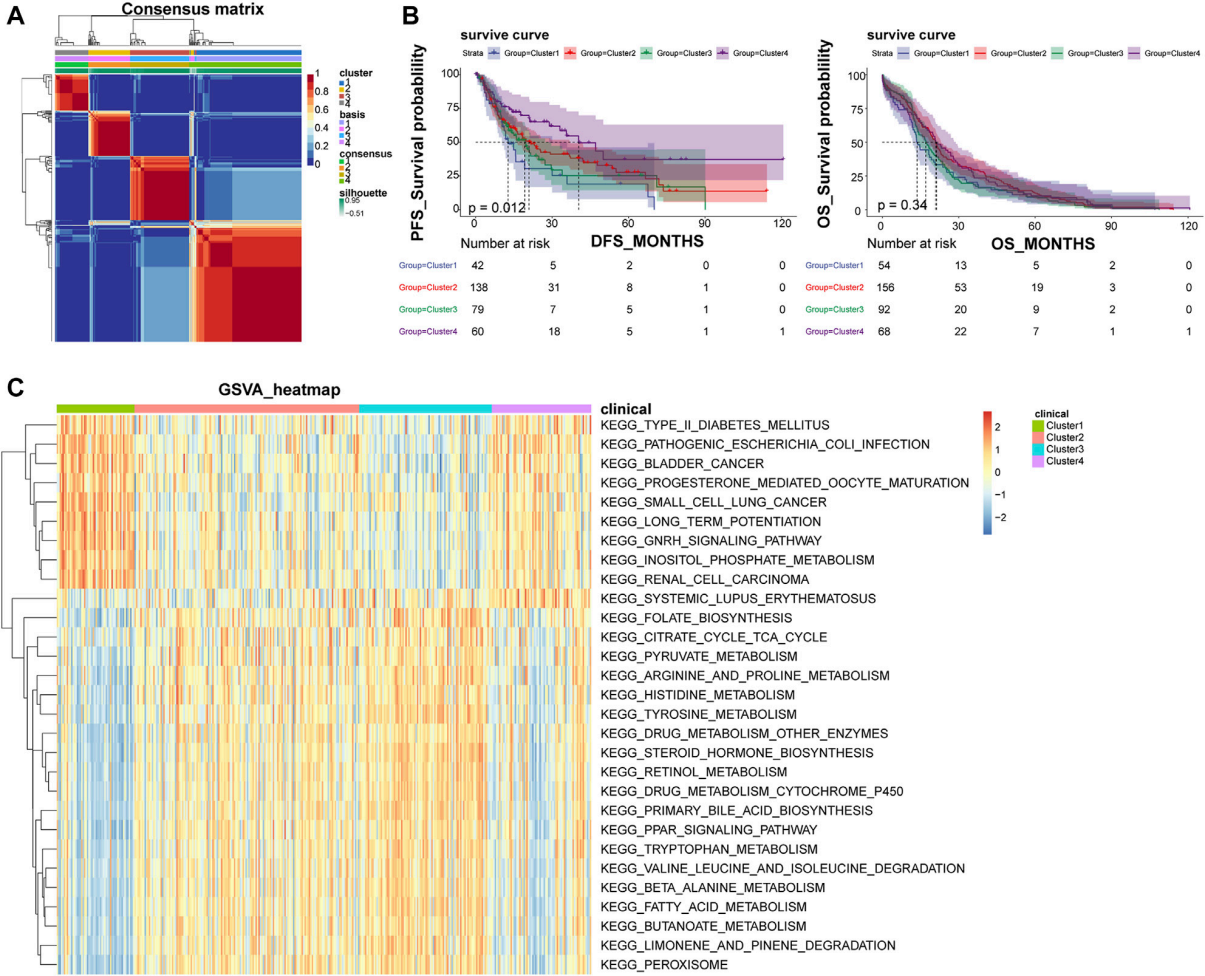
NMF clustering classify LIHC patients into four different subtypes. **(A)** Heat map of NMF clustering. **(B)** Survival curves of patients with different disease subtypes. The left panel shows the OS survival curve, and the right panel shows the DFS survival curve. **(C)** Heat map of four subtypes of GSVA enrichment pathways; NMF, negative matrix factorization; OS, overall survival; DFS, disease-free survival.

Based on the optimal rank value four of NMF clustering (Supplementary Figure S1A), the 371 LIHC patients were grouped into four subtypes, and their clustering heat map was shown in Figure 1A. Among them, Cluster one included 54 samples, Cluster 2 had 156 samples, Cluster three had 92 samples, and Cluster four had 69 samples. The expression of most of the cell adhesion molecules-related genes differed significantly among the subtypes, indicating a good discriminative effect of these genes on disease subtypes (Supplementary Figure S1B). In addition, survival analysis showed that overall survival (OS) did not differ significantly among subtypes, and disease-free survival (DFS) prognostic survival differed significantly, with Cluster one patients having the worst prognosis and Cluster four patients having the best prognosis (Figure 1B). A total of 29 KEGG pathways were enriched across subtypes, including some cancer-related metabolic pathways, such as small cell lung cancer, bladder cancer, renal cell carcinoma, and peroxisome proliferator-activated receptor signaling pathways, and alterations in these pathways may be important factors influencing the development of LIHC into different subtypes (Figure 1C).

### Significant differences in immune scores and immune cells among the different subtypes

**FIGURE 2:**
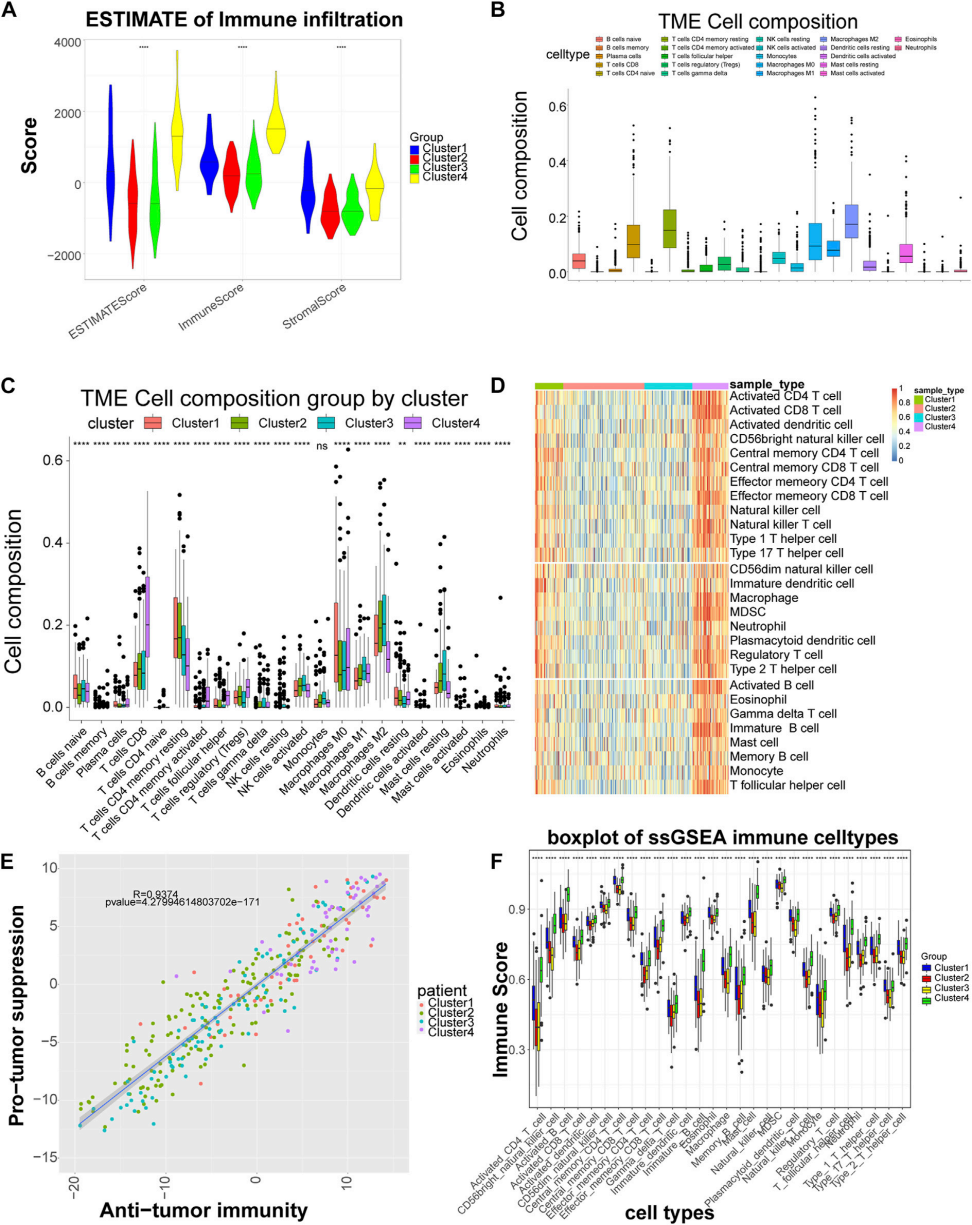
Significant differences in immune scores and immune cells among the different subtypes. **(A)** Distribution of the ESTIMATE score of each subtype. **(B)** Box plot of the proportional distribution of 22 immune cells in LIHC samples by CIBERSORT method. **(C)** Box plot of 22 immune cell distribution among four subtypes**. (D)** Heat map of immune cell distribution by ssGSEA method. The horizontal axis corresponds to different samples, and the vertical axis corresponds to different types of immune cells, with red representing high immunity and blue representing low immunity. **(E)** Correlation between tumor suppression and antitumor immunity. Anti-tumor immune score on the horizontal axis and anti-tumor score on the vertical axis. **(F)** Box plot of 28 immune cell among four subtypes. 28 cell types on the horizontal axis and immune scores on the vertical axis. **: *p* < 0.01,****: *p* < 0.0001, ns: no significant difference.

Stromal Score, Immune Score and ESTIMATE Score showed significant differences among subtypes, with Cluster four having the highest score, indicating that LIHC subtypes grouped based on cell adhesion molecules-related genes differed significantly in stromal cells, immune cells and tumor purity, with consistent trends (Figure 2A). The CIBERSORT algorithm found that among the 22 immune cells, except for macrophages, the distribution of the remaining 21 immune cells including B cells native, T cells CD8, etc. differed significantly between groups (Figures 2B,C). By ssGSEA analysis, the content of immune cells in Cluster four was significantly higher than other subtypes (Figure 2D). Also, in terms of organismal immune cell correlation, antitumor immunity was strongly correlated with tumor suppression (Figure 2E). In addition, ssGSEA analysis revealed that the distribution of 28 immune cells was different among subtypes (Figure 2F).

### DEGs among different subtypes were involved in the cancer-related pathway

**FIGURE 3:**
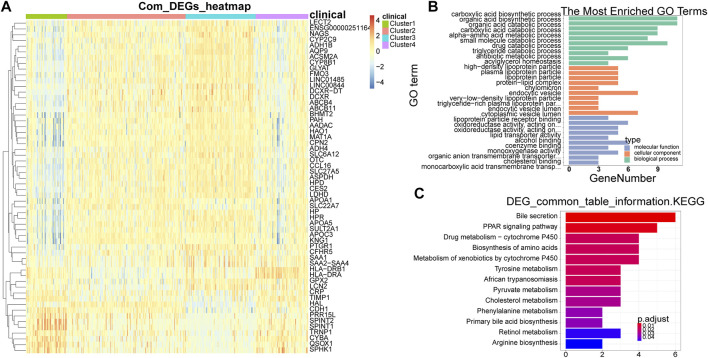
Identification and enrichment analysis of common DEGs. **(A)** Heat map of common DEGs among different subtypes. Each small square represents each gene, and its color indicates the expression level of the gene. The larger the expression level, the darker the color is (red is high expression, blue is low expression). **(B)** The most enriched GO terms are summarized. **(C)** Biological Processes bar plot. The ordinate represents each biological item, the abscissa represents the gene proportion, the color of the dot represents the *p*-value, the redder represents the higher confidence, the size of the dot represents the number of genes involved, and the larger the dot represents the more genes involved. DEGs, differentially expressed genes.

A total of 58 common DEGs were obtained by taking the intersection (Supplementary Table S1). The expression heat map of common DEGs among different subtypes was shown in Figure 3A, and the overall expression of common DEGs was significantly different among different subtypes. By enriching the functions and pathways of the common DEGs, they were mainly involved in the process of sugar and lipid metabolism and amino acid metabolic, and in the KEGG pathway, mainly involved in amino acid synthesis and sugar metabolism, in addition also involved in the cancer-related pathway, such as PPAR signaling pathway (Figures 3B,C).

### A risk model based on nine model genes was built

**FIGURE 4:**
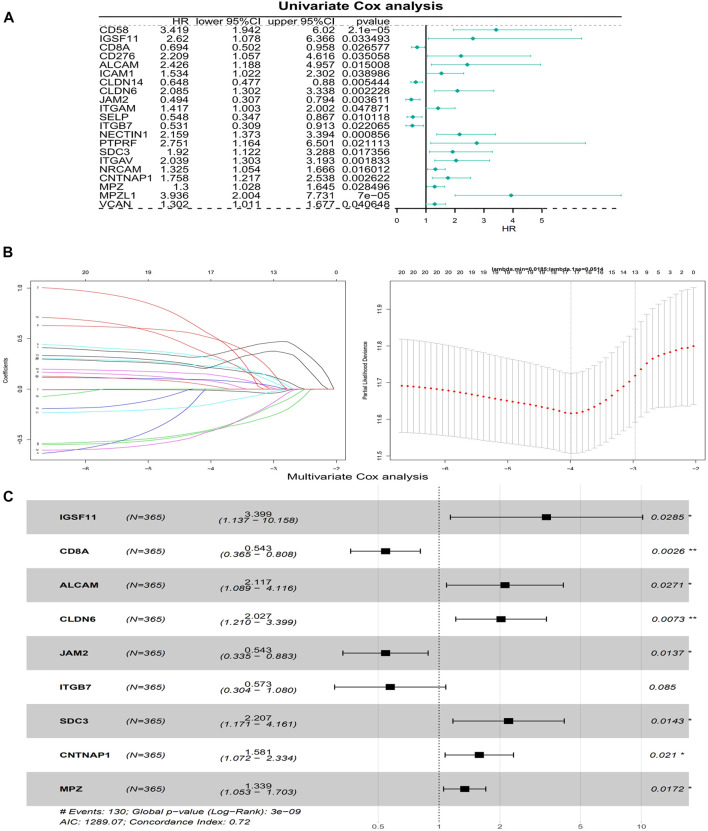
Building of a risk model. **(A)** A forest plot of Univariate Cox regression analysis. HR is the hazard ratio and Lower/Upper 95% CI is the 95% confidence interval of the value at risk. **(B)** Gene coefficient plot and cross-validation error plot of LASSO regression analysis. **(C)** A forest plot of Multivariate Cox regression analysis. HR is the hazard ratio and Lower/Upper 95% CI is the 95% confidence interval of the value at risk.

**FIGURE 5:**
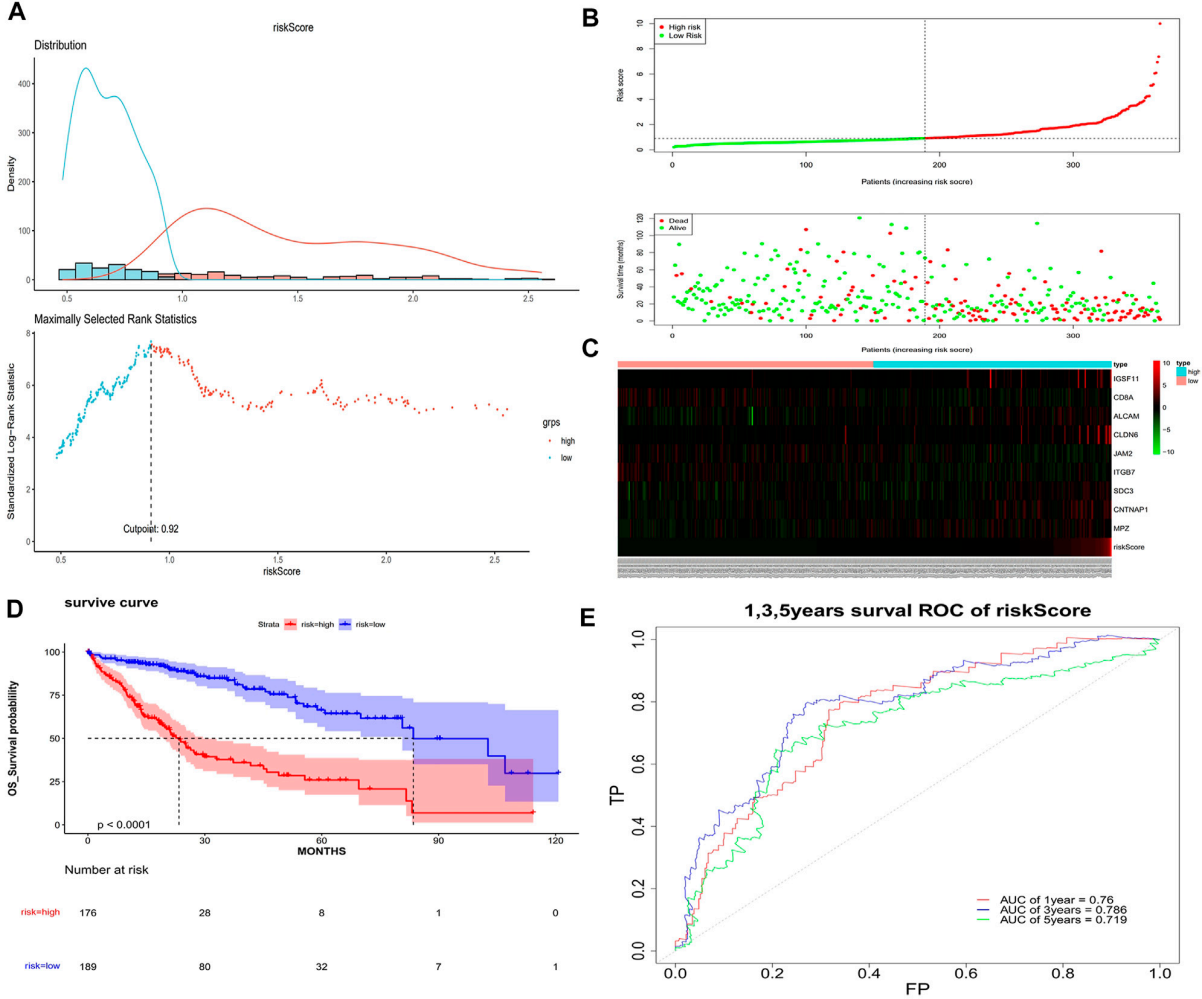
Assessment of the risk model. **(A)** Optimal threshold screening for risk distribution in the training set. **(B)** The risk score, survival time of patients in the training set. **(C)** The expression of model genes in the training set. **(D)** Survival curve of high- and low-risk patients in the training set. **(E)** ROC curves of the risk model in the training set.

**FIGURE 6:**
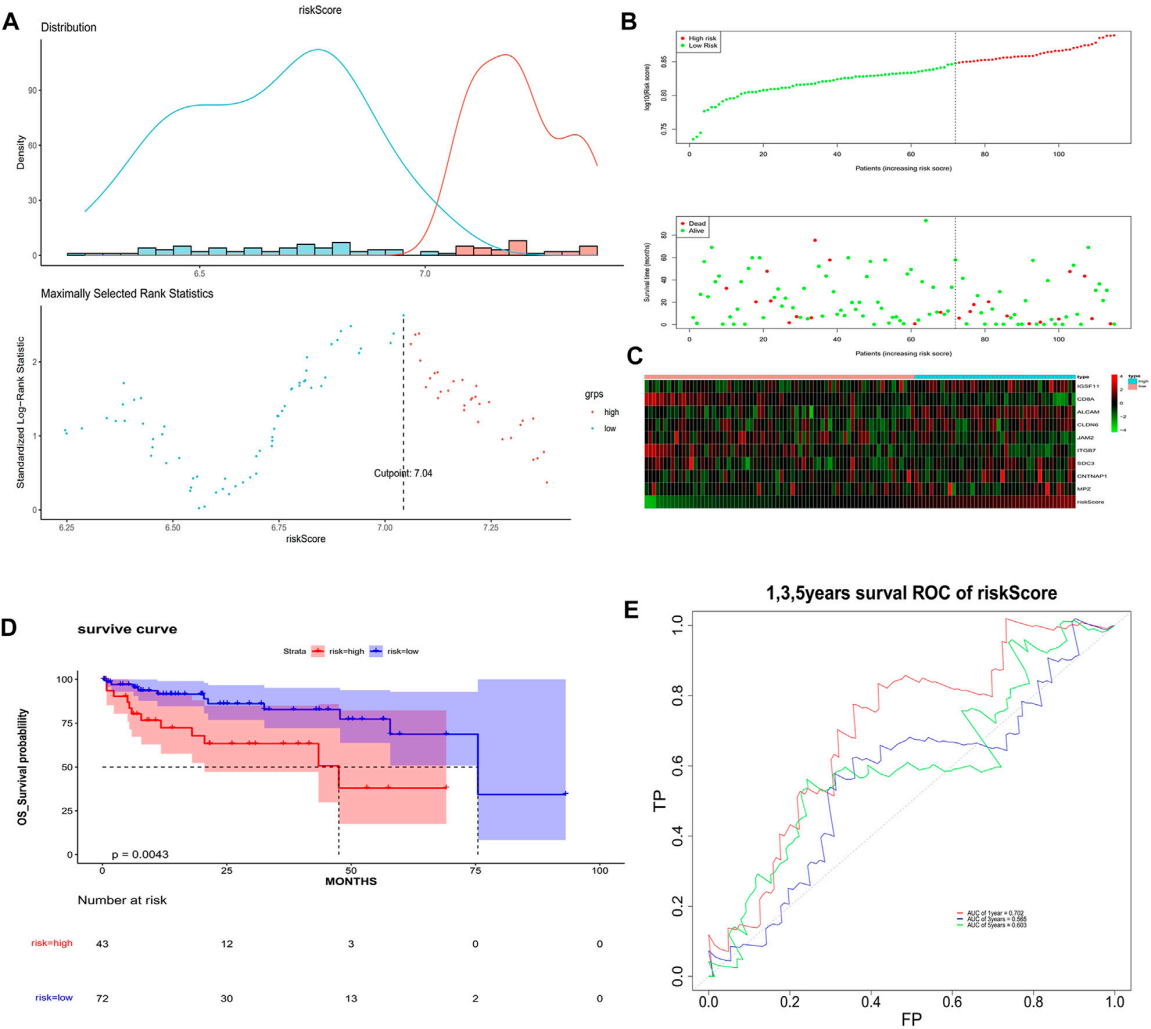
Validation of the risk model. **(A)** Optimal threshold screening for risk distribution in the validation set. **(B)** The risk score, survival time of patients in the validation set. **(C)** The expression of model genes in the validation set. **(D)** Survival curve of high- and low-risk patients in the validation set. **(E)** ROC curves of the risk model in the validation set.

The univariate Cox analysis yielded 21 candidate risk factors (Figure 4A; Table 2). A gene coefficient plot and a cross-validation error plot in the LASSO regression analysis were shown in Figure 4B. The best model was obtained when the penalty coefficient was equal to 0.0185 (lambda.min), and 17 characteristic genes were obtained, namely: CD58, IGSF11, CD8A, ALCAM, ICAM1, CLDN14, CLDN6, JAM2, SELP, ITGB7, NECTIN1, SDC3, NRCAM, CNTNAP1, MPZ, MPZL1, and VCAN. Multivariate Cox analysis yielded nine model genes, including IGSF11, CD8A, ALCAM, CLDN6, JAM2, ITGB7, SDC3, CNTNAP1, and MPZ (Figure 4C). The differences in the expression of nine model genes in 4 clusters were displayed in Supplementary Figure S2.365 patients were grouped into high (n = 176) and low risk groups (n = 189) with the optimal threshold (Figure 5A). Supplementary Figure S3 exhibited the patients’ distribution in four clusters, different survival status, and two risk groups. The risk curves were shown in Figure 5B, and the expression of model genes was shown in Figure 5C. In addition, the OS of patients in the high-risk group was lower, which was consistent with the actual, which indicated that the constructed risk model was in accordance with the theoretical facts (Figure 5D). The area under curves (AUCs) of 1, 3, and 5 years in the ROC curves were 0.76, 0.786, and 0.719, respectively, with the highest diagnostic efficiency for the 3-year survival prognosis (Figure 5E). The risk curves of the validation set were shown in Figure 6A with the optimal threshold (Figure 6B). In addition, the OS of patients in the low-risk group was longer (Figure 6C), and the AUC values of ROC curves were >0.6 (Figure 6D). Moreover, the expression of model genes was largely consistent with that of the training set (Figure 6E).

**TABLE 2:** The results of univariate Cox analysis generated by univariate Cox analysis.

Gene	HR	HR.95L	HR.95H	*p*-value
CD58	3.419	1.942	6.02	2.10E-05
IGSF11	2.62	1.078	6.366	0.033
CD8A	0.694	0.502	0.958	0.027
CD276	2.209	1.057	4.616	0.035
ALCAM	2.426	1.188	4.957	0.015
ICAM1	1.534	1.022	2.302	0.039
CLDN14	0.648	0.477	0.88	0.005
CLDN6	2.085	1.302	3.338	0.002
JAM2	0.494	0.307	0.794	0.004
ITGAM	1.417	1.003	2.002	0.048
SELP	0.548	0.347	0.867	0.010
ITGB7	0.531	0.309	0.913	0.022
NECTIN1	2.159	1.373	3.394	0.001
PTPRF	2.751	1.164	6.501	0.021
SDC3	1.92	1.122	3.288	0.017
ITGAV	2.039	1.303	3.193	0.002
NRCAM	1.325	1.054	1.666	0.016
CNTNAP1	1.758	1.217	2.538	0.003
MPZ	1.3	1.028	1.645	0.028
MPZL1	3.936	2.004	7.731	7.00E-05
VCAN	1.302	1.011	1.677	0.040

### Independent prognostic analysis and building of a nomogram

**FIGURE 7:**
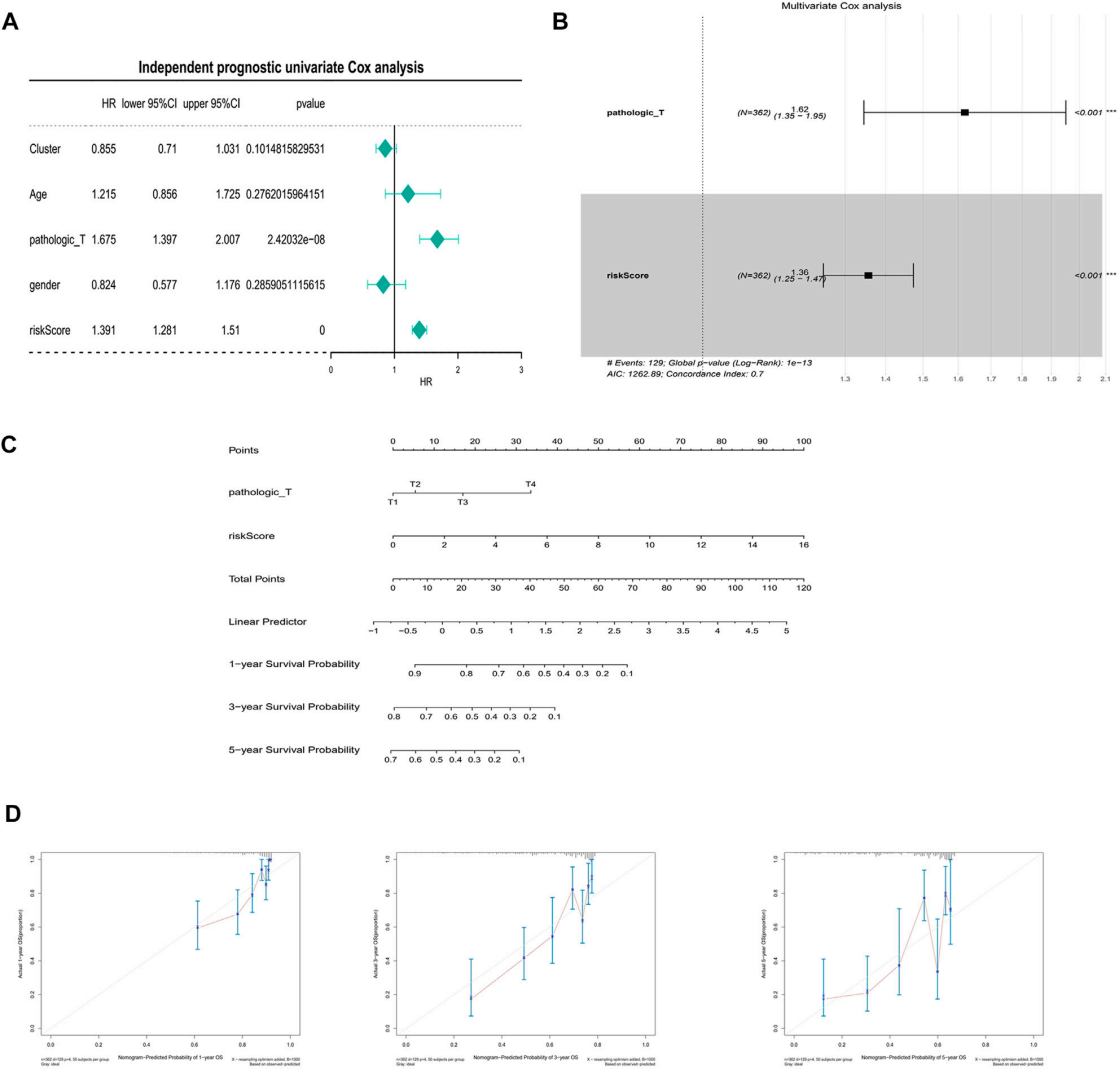
Independent prognostic analysis and building of a nomogram. **(A)** Univariate Cox independent prognostic analysis in the training set. **(B)** Multivariate Cox independent prognostic analysis in training set. **(C)** A nomogram predicting overall survival for HCC patients. For each patient, three lines are drawn upward to determine the points received from the three predictors in the nomogram. The sum of these points is located on the ‘Total Points’ axis. Then a line is drawn downward to determine the possibility of 1-, 3-, and 5-year overall survival of HCC. **(D)** The calibration plot for internal validation of the nomogram. The *Y*-axis represents actual survival, and the *X*-axis represents nomogram-predicted survival.

In the univariate Cox analysis, the *p*-values of pathologic T and riskScore were less than 0.05, and the HR values were greater than 1, which were risk factors (Figure 7A). The multivariate Cox results showed that pathologic T, riskScore were highly significant in the prognostic model with an AIC value of 1,262.89 and a diagnostic *p*-value of 1e-13 (Figure 7B). The predictive effect of the combination of pathologic T and riskScore on the prognostic survival of patients at 1, 3, and 5 years can be visualized by the Nomogram (Figure 7C). The calibration curve showed that the predicted values of survival at 1, 3, and 5 years were generally consistent with the actual, i.e., they fluctuated around the diagonal, indicating that the Nomogram has good predictive value (Figure 7D).

### GSEA between high- and low-risk groups

**FIGURE 8:**
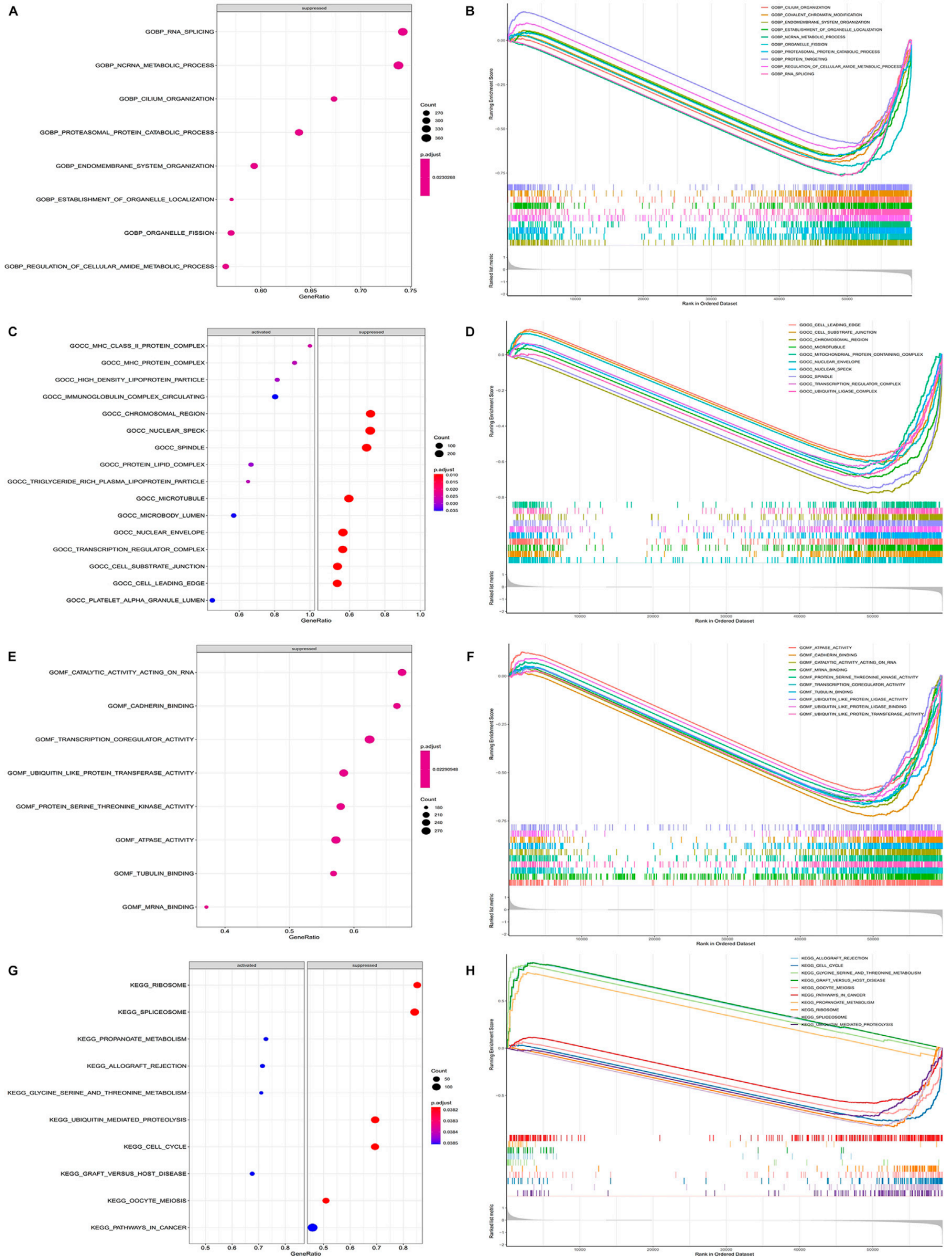
Functional enrichment of GSEA between high and low risk groups. **(A)** Plot of GSEA rich distribution points between high and low risk groups (GO-BP). The vertical axis is the functional item, the horizontal axis is the gene proportion, the left figure is the pathway activated by a single gene, the right figure is the pathway suppressed by a single gene, the size of the dot is proportional to the number of genes, and the color is related to significance. **(B)** GSEA enrichment trend map between high and low risk groups (GO-BP). **(C)** Plot of GSEA rich distribution points between high and low risk groups (GO-CC). **(D)** GSEA enrichment trend map between high and low risk groups (GO-CC). **(E)** Plot of GSEA rich distribution points between high and low risk groups (GO-MF). **(F)** GSEA enrichment trend map between high and low risk groups (GO-MF). **(G)** GSEA Rich distribution plot between high and low risk groups (KEGG). The vertical axis is the functional item, the horizontal axis is the gene proportion, the left figure is the pathway activated by a single gene, the right figure is the pathway suppressed by a single gene, the size of the dot is proportional to the number of genes, and the color is related to significance. **(H)** GSEA enrichment trend map between high and low risk groups (KEGG).

The GSEA showed that the biological processes involved RNA splicing, ncRNA metabolic process, endomembrane system organization, *etc.* (Figures 8A–F). The metabolic pathways involved mainly included cell cycle, pathways in cancer, both of which were closely related to the development of cancer (Figures 8G,H). This suggested that the model genes may be directly or indirectly involved in these cancer-associated pathways to influence the prognostic between patients.

### qRT-PCR explored the expression of model genes

**FIGURE 9:**
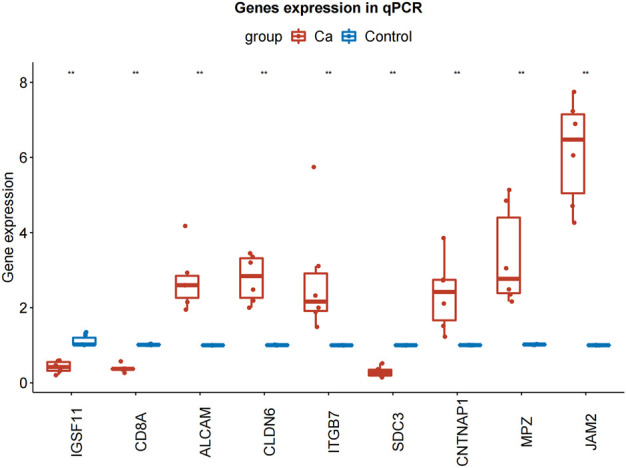
qRT-PCR results of IGSF11, CD8A, ALCAM, CLDN6, JAM2, ITGB7, SDC3, CNTNAP1, and MPZ in LIHC and normal samples. qRT-PCR, quantificational rt-PCR.

To further explore the expression of model genes, we used qRT-PCR to compare the expression levels of IGSF11, CD8A, ALCAM, CLDN6, JAM2, ITGB7, SDC3, CNTNAP1, and MPZ in LIHC tissue and paracancerous tissue. Compared with normal tissue, the expression of ALCAM, CLDN6, ITGB7, CNTNAP1, JAM2, and MPZ in LIHC tissue was significantly up-regulated, the expressions of IGSF11, CD8A, and SDC3 in LIHC tissue were significantly down-regulated (Figure 9; Table 3).

**TABLE 3:** The qRT-PCR result of model genes in cancer and normal samples.

	NC	Ca	T, df	*p*-value
IGSF11	1.1321 ± 0.1617	0.4137 ± 0.1728	t = 6.434, df = 5	0.0013
CD8A	1.0197 ± 0.0146	0.3909 ± 0.1122	t = 14.61, df = 5	<0.0001
ALCAM	1.0017 ± 0.0011	2.8922 ± 0.7698	t = 5.385, df = 5	0.003
CLDN6	1.0055 ± 0.0068	2.6467 ± 0.6044	t = 6.910, df = 5	0.001
ITGB7	1.0024 ± 0.0015	2.1623 ± 0.6078	t = 2.758, df = 5	0.0399
SDC3	1.0021 ± 0.0016	0.2782 ± 0.1489	t = 12.65, df = 5	<0.0001
CNTNAP1	1.0056 ± 0.0033	2.2928 ± 1.0503	t = 3.481, df = 5	0.0176
MPZ	1.0203 ± 0.0093	3.3990 ± 1.4629	t = 4.328, df = 5	0.0075
JAM2	1.0033 ± 0.0029	6.0010 ± 1.5205	t = 8.944, df = 5	0.0003

NC, normal control; Ca, cancer.

## Discussion

LIHC is the fourth leading cause of cancer-related death worldwide (Yang et al., 2019), and it and it is still a major challenge facing global public health. Although clinical routine parameters such as T, N, M staging can help evaluate the prognosis of patients to a certain extent, the current prediction methods still fail to evaluate the prognosis of patients efficiently and comprehensively due to the complex molecular mechanism and great heterogeneity of its pathogenesis. Therefore, it is urgent to explore new biomarkers to evaluate the prognosis and establish a more accurate prognosis model.

Cell adhesion molecules are mainly transmembrane receptor proteins, which maintain cell-cell contact and adhesion to extracellular matrix, but they are also signal effector molecules involved in cell functions, such as cell growth, survival, and transcriptional activity (Windisch et al., 2019). In this study, we performed consistent cluster analysis on LIHC patients based on the expression of adhesion molecule signaling pathway related genes combined with algorithm for the first time and found four subtypes of LIHC. Through the analysis of the body immunity among various subtypes, we know that the fourth liver cancer subtype based on the classification of cell adhesion molecule related genes has the highest score in terms of stromal cells, immune cells and tumor purity, and its immune cell content is the highest, especially cd8+ T cell, the proportion of the fourth subtype is much higher than that of the other three groups. Previous studies have shown that the effect of immunotherapy was positively correlated with the degree of tumor infiltration of CD8 T cells (Durgeau et al., 2018). Wang showed that CD8 + T cells were crucial for the formation of anti-tumor immunity in LIHC, and their increased infiltration was related to a good prognosis (Wang et al., 2022). CD8T cells play a key role in the elimination of intracellular infections and malignant cells and can provide long-term protective immunity. The main functional subgroup of CD8T cells iscytotoxic T lymphocytes (CTL) that directly kill tumor cells. Immune suppressive factors of the tumor microenvironment (TME) undermine viability and exhaust the activities of the intratumoral CD8 T lymphocytes thereby evading anti-tumor immunity and decreasing the benefits of immune therapies (Lu et al., 2022). So the cluster four of patients may had a significant response to antitumor immunotherapy. David Y’s study showed that CD4T cells can also mediate cytotoxicity in cancer should lead to novel approaches to further enhance their direct anti-tumor activity in patients (Oh and Fong 2021). CD4^+^ T cells can provide “help” by recruiting CD8 T cells, increasing their proliferation, and enhancing their effector function through IFN-*γ*-dependent production of chemokines and IL-2 (Wang et al., 2020). However, Regulatory T (Treg) cells maintain immune homeostasis by inhibiting abnormal/overactive immune responses to both autogenic and nonautogenic antigens. Treg cells have two functional characteristics: T cell anergy and immunosuppression. In the process of tumor development, Treg cells accumulate locally in the tumor and lead to tumor escape by inducing anergy and immunosuppression (Chen B. J. et al., 2022). We can see that in both tumor microenvironment and ssGSEA analysis, the proportion of Tregs in group 4 is higher than that in the other three groups, possibly because the proportion of CD8 cells is higher, which activates the effect of Tregs. Therefore, inhibition/clearance of Treg cells is a promising strategy for enhancing antitumor immunity in cluster 4. This finding also provides a high specific prospect for personalized treatment of liver cancer among different subtypes. Meanwhile, previous studies have shown that cell adhesion molecules also mediate tumor metastasis, Interactions between disseminated tumor cells (DTC) and stromal cells in the microenvironment are critical for tumor colonization of distal organs (Chen and Massague 2012). Sharma R provided that vascular cell adhesion molecule-1 (VCAM-1) has been found to be involved in this process. VCAM-1 is aberrantly expressed in breast cancer cells, and that it can bind to its natural ligand α4β1integrin, also denoted as very late antigen 4 (VLA-4). This binding appears to be responsible for the metastasis of breast cancer cells to lung, bone and brain. And this research represents a potential therapeutic target for metastatic breast cancer (Sharma et al., 2017). Yuan showed that TXNDC12 is frequently upregulated in HCC, particularly in metastatic lesions, suggesting that TXNDC12 may promote HCC metastasis (Yuan et al., 2020). These studies indicate that cell adhesion molecules are involved in tumor metastasis either in the forward or the reverse direction, and also provide a new direction for future research.

At the same time, by analyzing the differences between four groups, 58 common DEGs were screened, and their functional pathways were enriched. It was found that they participated in the cancer-related pathway - PPAR signaling pathway. Peroxisome proliferator activated receptor (PPAR) is a nuclear receptor and transcriptional regulator, which plays a key role in liver and systemic energy homeostasis (Berthier et al., 2021). Zuo indicated Low levels of PGC1α expression indicate a poor prognosis for LIHC patients. PGC1α suppresses LIHC metastasis by inhibiting aerobic glycolysis through regulating the WNT/β-catenin/PDK1 axis, which depends on PPARγ. PGC1α is a potential factor for predicting prognosis and a therapeutic target for LIHC patients. Xu established a PPAR related polygenic model that can be reliably used as an independent predictor of LIHC prognosis (Xu et al., 2021). These studies have shown that PPAR pathway was closely related to the occurrence, development and prognosis of LIHC.

In this study, we established a prognostic diagnostic model based on cell adhesion genes, which is composed of IGSF11, CD8A, ALCAM, CLDN6, JAM2, ITGB7, SDC3, CNTNAP1, MPZ. According to the expression of these genes and the coefficient of the prognostic model. In recent years, studies have also established some models to predict the survival and prognosis of LIHC patients. Liu have established a prognostic risk model composed of six genes, which has become an effective indicator to evaluate the survival and prognosis of LIHC patients (Liu et al., 2019). However, our research is based on cell adhesion molecules, which is more detailed than the previously mentioned research, and we have made PCR external verification, so the model is more persuasive. Zhao established a prognostic risk model of nine amino acid metabolism related genes in LIHC. The model predicts the overall survival rate of LIHC patients based on amino acid metabolism related genes. However, the AUC value of 3-year and 5-year survival rates of this model is lower than our model (Zhao et al., 2021).

Immunoglobulin superfamily 11 gene (IGSF11) is a 46KD protein containing 431 amino acids, which is located on chromosome 3q13.32 and acts on cell adhesion, migration, proliferation and differentiation. Xiyang Tang’s research shows that IGSF11 and Vista are a pair of immune checkpoints, which act on tumor proliferation and immune regulation, and have great potential as new tumor immunotherapy targets and biomarkers. Targeting IGFS11 is suitable for the treatment of colorectal cancer and hepatocellular carcinoma outside intestinal gastric cancer. It is an ideal target for cancer immunotherapy (Watanabe et al., 2005). CD8a expression can be detected in natural killer cells and dendritic cells, although CD8a is mainly expressed on the surface of cytotoxic T cells. CD8 positive cytolytic T cells (CD8ctl) play a crucial role in the cellular immune system and cell-mediated immune responses. Guo said that in osteosarcoma disease, the high CD8a expression group has a better survival probability than the low CD8a expression group, which belongs to the protective gene (Guo et al., 2021). Activated leukocyte adhesion molecule (ALCAM) is a glycoprotein involved in the adhesion of homotypic and heterotypic cells. ALCAM can perform proteolytic cleavage on the cell surface by metalloproteinase, which produces the abscission of its ectodermal structure. In a variety of cancers such as laryngeal squamous cell carcinoma, ALCAM overexpression can be used as an important prognostic marker of disease progression (Ferragut et al., 2021). ALCAM gene overexpression in laryngeal squamous cell carcinoma was poor. Claudins (CLDNs) are the most important tight junction proteins, which are mainly expressed in endothelial cells or epithelial cells in a tissue-specific manner (Qu et al., 2021). Reactivation of CLDN6 is often observed in LIHC tumor tissues and precancerous lesions. Functional tests showed that CLDN6 is not only a tumor associated antigen, but also has a strong carcinogenic effect in LIHC(Kong et al., 2021). After CLDN6 silencing, the proliferation, migration and invasion of LIHC cells were inhibited. JAMs have multiple functions that include regulation of endothelial and epithelial paracellular permeability, leukocyte recruitment during inflammation, angiogenesis, cell migration and proliferation (Luissint et al., 2014). According to the research of Zhao, the expression of JAM-2 in colon cancer cell line can reduce the growth, adhesion, migration and invasion of tumor cells (Zhao et al., 2016). The research of Yang shows that the overexpression of JAM-2 can block the invasion and migration of breast cancer cells. The expression level of JAM-2 in breast cancer is low, and the prognosis of patients with high expression of JAM-2 is good. JAM-2 has good clinical diagnostic and prognostic value (Peng et al., 2022). Due to the lack of relevant literature on JAM-2 gene, its specific role in liver cancer needs further study. Integrin β7 (ITGB7), which is expressed on the surface of leukocytes, plays an essential role in the homing of immune cells to gut-associated lymphoid tissue and facilitating the retention of lymphocytes in gut epithelium (Zhang et al., 2021). Chen’s research ITGB7 has a significant correlation with the tumor microenvironment of hepatocellular carcinoma, may represent new hepatocellular carcinoma ferroptosis inducing markers and have guiding significance for the treatment of hepatocellular carcinoma (Chen Y. et al., 2022). The syndecan (SDC) family consists of four transmembrane type I proteoglycans, SDC3 is expressed primarily by neuronal tissue and cartilage. Nilton Jos é Santos found that patients with SDC3 immunostaining positive prostate cancer had a poor prognosis (Santos et al., 2021). CNTNAP1 (contactin-associated protein 1, also known as paranodin), a single-pass transmembrane protein, was originally identified in neurons by its interaction with contactin protein, plays a role in the production and maintenance of accessory lymph nodes in myelin axons (Zhao et al., 2018). Myelin protein zero (MPZ) is a member of the immunoglobulin gene superfamily with single extracellular, transmembrane and cytoplasmic domains (Shy et al., 2004). At present, most of the existing articles on MPZ are in neuropathy, and there are few studies related to liver cancer, which need to be further explored.

In addition, according to the classification of patients in high-risk and low-risk groups, we enriched the GSEA functional pathway between the two groups. The results showed that the DEGs between the two groups were involved in cancer-related pathways. It is suggested that the model gene composed of nine cell adhesion genes may directly or indirectly participate in these cancer-related pathways, thereby affecting the prognosis of patients. To sum up, in this study, on the one hand, liver cancer patients are clustered into four subtypes through the expression of cell adhesion related genes. There are significant differences in biological processes and pathways between these subtypes. At the same time, there are also significant differences in the immune microenvironment of the body, indicating that cell adhesion genes have important diagnostic value for the search of liver cancer subtypes and can be used as diagnostic markers. Besides, we have verified the differential expression of the 9-prognostic genes among the different clusters, we can find that the expressions of ALCAM, CLDN6, CNTNAP1, JAM2, SDC3 were significantly increased in cluster 1, the expressions of CD8A and ITGB7 were significantly increased in cluster 4, the expression of MPZ was significantly increased in cluster 3. Overexpression of JAM2 can block the invasion and migration of breast cancer cells, and the mechanism may be that JAM2 inhibits the Epithelial-Mesenchymal Transition pathway (Peng et al., 2022). JAM2 as a potential marker for a subfraction of HSCs with an extensive lymphopoietic capacity, mainly in T lymphopoiesis (Radulovic et al., 2019). However, the study of JAM2 in hepatocellular carcinoma is less, and it may lead to a good prognosis in cluster one patients through these above approaches. ALCAM has previously been shown to be involved in tumor progression and metastasis (Wai et al., 2012). the recognition that ALCAM may serve as a pivotal receptor for a cancer cell to seek its metastatic destination places it as an important player in the ‘seed and soil’ theory of cancer metastasis proposed more than a century ago (Paget 1989). Many studies have found that ALCAM has been shown to be highly expressed in various tumors, therefore, targeted inhibition of ALCAM may become a new idea for the treatment of cluster 1. At the same time, CD8 was proved to be significantly increased in cluster 4, which can be combined with the tumor immune microenvironment discussed above as the new treatment idea for this type in LIHC. On the other hand, based on the expression of cell adhesion related genes, combined with the prognosis and survival of patients, prognostic molecular diagnostic models are obtained. These model genes have good diagnostic significance for the prognosis of patients and provide an important reference for the molecular diagnosis of the prognosis of patients in the future. However, several limitations of our study should be taken into consideration. First, the model was established with tumour tissues, so it can only predict the prognosis of LIHC patients after surgery and cannot detect and diagnose tumours at the early stage. And then, further functional experiments are needed, and the underlying mechanism of the nine genes needs to be clarified.

## Conclusion

In this study, we established a novel nine-gene (IGSF11, CD8A, ALCAM, CLDN6, JAM2, ITGB7, SDC3, CNTNAP1, MPZ) prognostic diagnostic model based on cell adhesion genes, which may provide a new idea for predicting the prognosis of clinical liver cancer patients.

## Data Availability

The datasets presented in this study can be found in online repositories. The names of the repository/repositories and accession number(s) can be found in the article/Supplementary Material.
