# Supplementary figures and images for: Identification of subtypes of hepatocellular carcinoma and screening of prognostic molecular diagnostic markers based on cell adhesion molecule related genes
Source: Front Genet. 2022 Nov 22;13:1042540. doi: 10.3389/fgene.2022.1042540 (PMC9723242; doi:10.3389/fgene.2022.1042540)

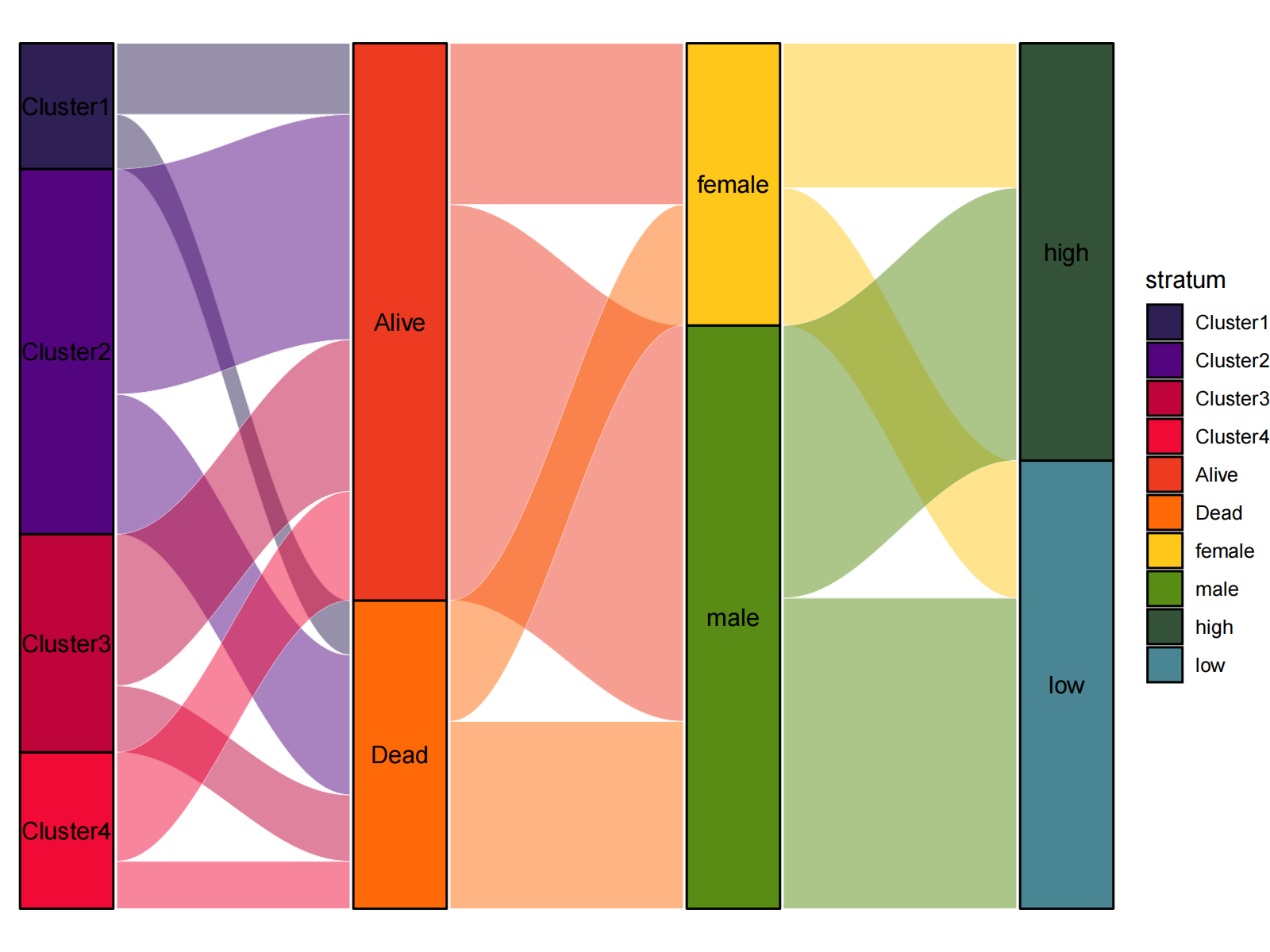

Supplement: Supplementary file 1 [file Image3.TIF]

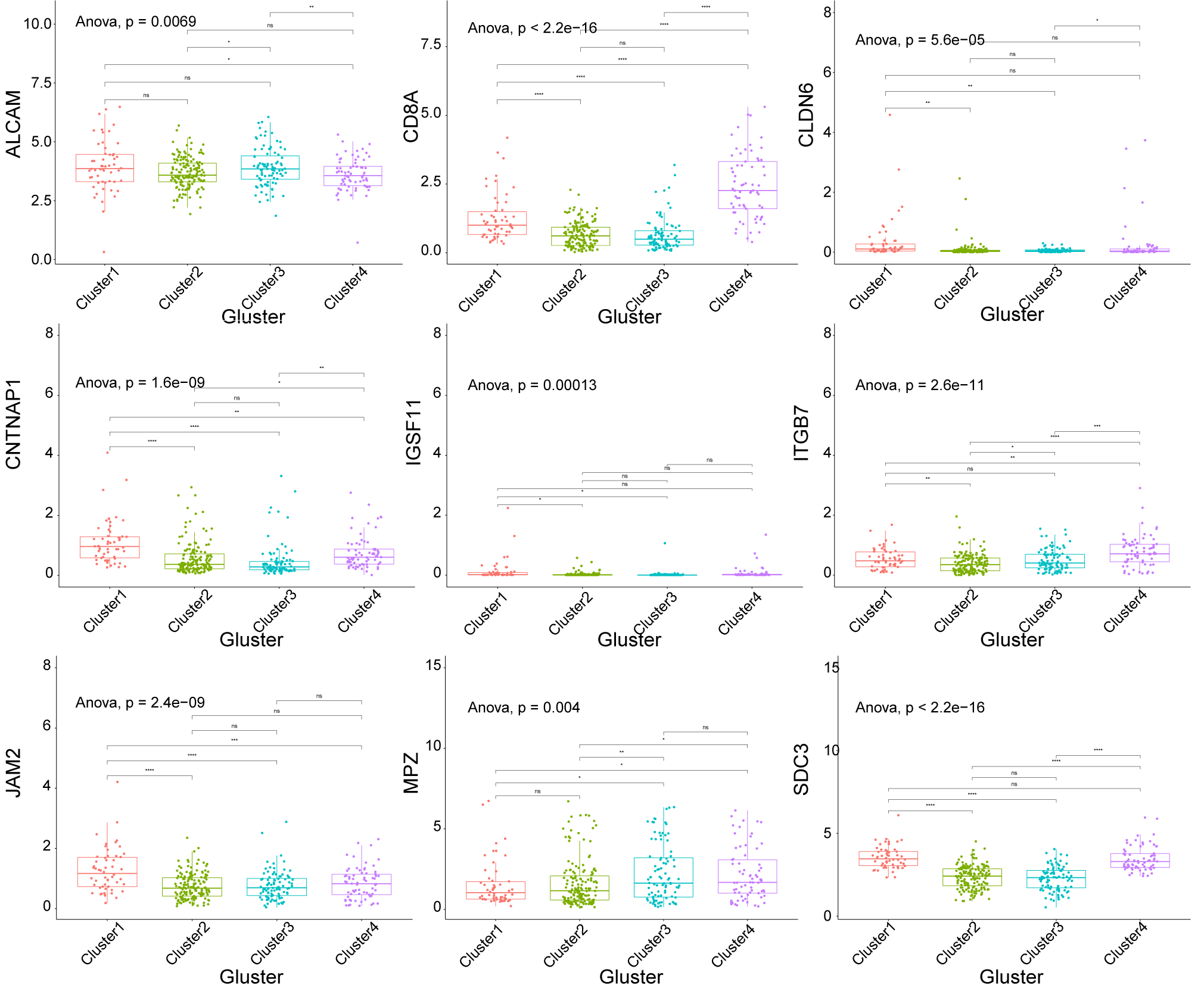

Supplement: Supplementary file 2 [file Image2.TIF]

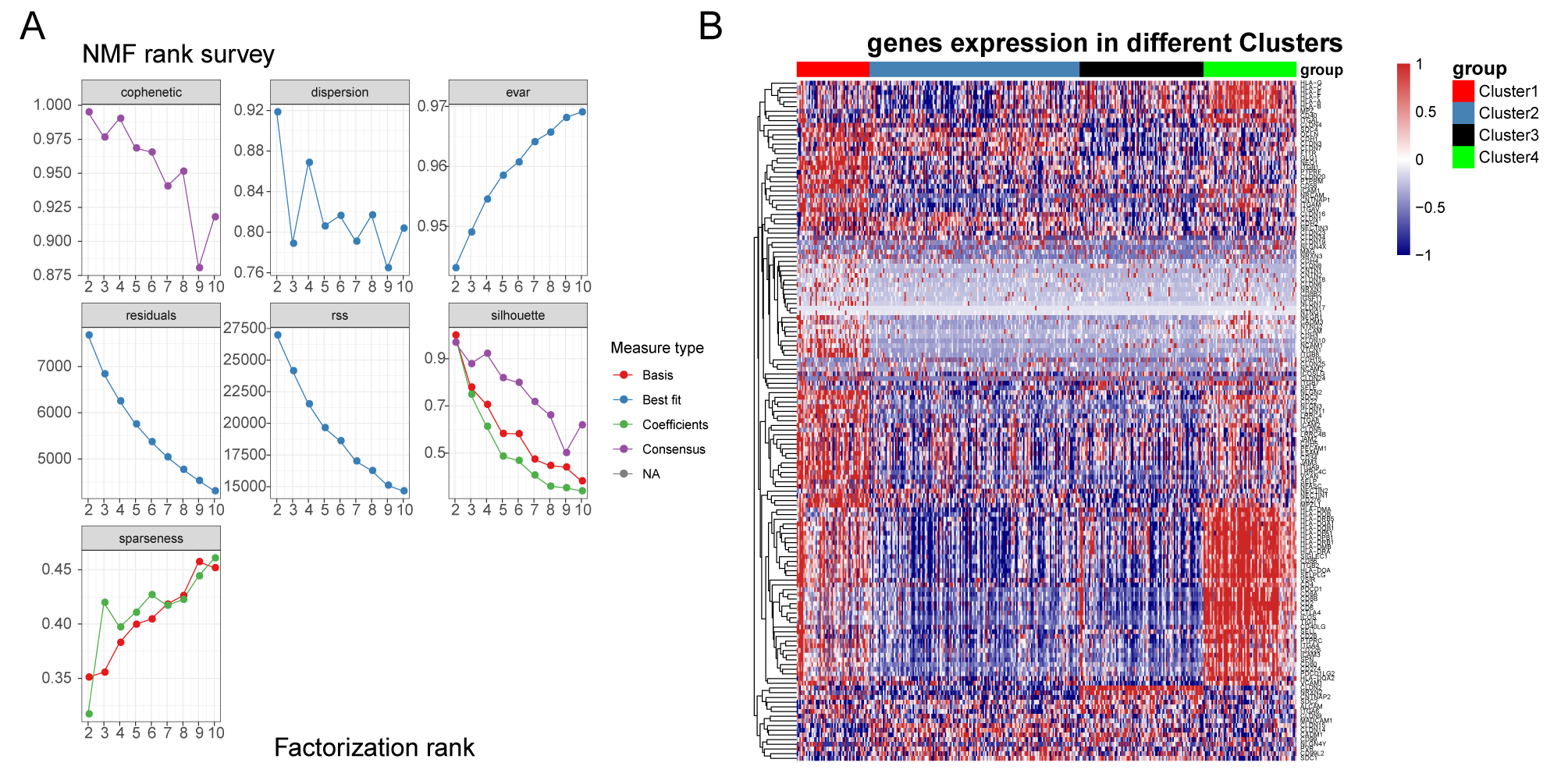

Supplement: Supplementary file 3 [file Image1.TIF]
